# Supplementary material for: Patterns of Intron Gain and Loss in Fungi
Source: PLoS Biol. 2004 Nov 30;2(12):e422. doi: 10.1371/journal.pbio.0020422 (PMC532390; doi:10.1371/journal.pbio.0020422)
Supplement: Table S1 — Also available at http://genes.mit.edu/NielsenEtAl/. (4.3 MB ZIP). [file pbio.0020422.st001.zip › NielsenEtAl/html/1186.html]

AN8863.1.NCU01438.1.MG06924.1.FG07467.1


```
 CLUSTAL W (1.82) Multiple Sequence Alignments - Introns Inserted


Sequence 1: MG06924.1	404 aa
Sequence 2: FG07467.1	406 aa
Sequence 3: NCU01438.1	411 aa
Sequence 4: AN8863.1	394 aa
Alignment Length: 420 aa
Number Identitical Residues: 252 aa
Alignment Score (without introns) 11256


MG06924.1 	MAEPIPNKRAGADPIAP2----TPQNTPAQHAPITSHAQQPGVDSIKEE1DFT---AASI
NCU01438.1	MAEPIRNKRA-DPPIAP2LRFSTPQNTPLNTAPISSHAQQPGVASIKEE1DLDRAAAASI
FG07467.1 	MAEPIRGKRA-QDAVAP2----TPQNTPATAAPISSHAQQPGVASIKEE1DLDRAAAASV
AN8863.1  	MSEPIRNKKA-DFPVAP2----TPQNTPANNAPISSHAQQPGVSSIKEE1SLDHATAASL
          	*:*** .*:*   .:**     ******   ***:******** ***** .:  ::***:

MG06924.1 	FAQNPKLVQMIQGRLGTLVGRSSGYIESLPPQVKRRVAGLKGIQKEHSKLEAEFQEEVLE
NCU01438.1	FAKDPRLVSLIQGRLGSLVGRSSGYIESLPTDVKRRVAGLKGIQKEHSKLEAEFQEEVLQ
FG07467.1 	FAQNPKLIQMIQGRLGSLVGQSSGYIESLPAPVRRRVAGLKGIQKDHSKLEAEFQEEVLQ
AN8863.1  	FARNPGLVSMIQGKLGSLVGRSSGYIESLPVSVRRRVAGLKGIQKEHAKLEAQFQEEVLE
          	**::* *:.:***:**:***:*********  *:***********:*:****:******:

MG06924.1 	LEKKYFAKFTPLYQKRSAIVNGSNEPTESEIEAGEESDEVDEEGEKKEVKTEETAASEAS
NCU01438.1	LEKKYFAKFTPLYQQRAAIVNGQTEPTEDQVQAGEEDEE-EEDEETEAAPQEEKATESAE
FG07467.1 	LEKKYFAKFSPLYEKRSAIINGKSEPTEDEIKAGEEDD--EEVDDDDAATKSETKSDVPD
AN8863.1  	LEKKYFAKFTPLYERRATIVNGATEPTETEIEAGKGEEE-EIDAKDEDLKQGE--DEKES
          	*********:***::*::*:** .**** :::**: .:. :   . .     *   .  .

MG06924.1 	DVKGIPEFWLSAMKNQISLAEMITDRDEQALKHLTDIRMEYLDKPGFRLIFEFSENEFFS
NCU01438.1	KVSGIPEFWLSAMKNQISLAEMITDRDEEALKSLTDIRMEYLDKPGFRLIFEFAENEYFT
FG07467.1 	NVSGIPEFWLSAMKNQISLAEMITDRDEAALKHLTDIRMEYLDKPGFRLIFEFAPNDFFS
AN8863.1  	AVTGIPEFWLSAMKNQISLAEMITDKDEEALKHLIDIRMEYLDRPGFRLIFEFSENEFFT
          	 *.**********************:** *** * ********:*********: *::*:

MG06924.1 	NKTISKTYYYQNESGYGGDFIYDHAEGDKIEWKGDDKDLT~VRVEQKKQRNKN1TKQTRI
NCU01438.1	NKTITKTYYYQNESGYGGDFIYDHAEGDKINWK-EDKDLT~VRVEQKKQRNKT1TKQTRI
FG07467.1 	NTTITKTYYYQNESGYGGDFIYDHAEGDKIDWS-PGKDLT~VRVESKKQRNKN1TKQTRI
AN8863.1  	NKTISKTYFYKEENGYGGDFIYDHAEGTKIDWK-PEKDLT0------------~---TRV
          	*.**:***:*::*.************* **:*.   ****                 **:

MG06924.1 	VKKTVPTESFFNFFSPPKAPTEDDD-DAASDIEDRLELDYQLGEDIKEKLIPRAIDWFTG
NCU01438.1	VKKTVPTESFFNFFSPPKAPTDDDE-STTSDIEERLELDYQLGEDIKEKLIPRAIDWFTG
FG07467.1 	VKKTVPTESFFNFFSPPKAPTDDDDEDAESDIEERLELDYQLGEDIKEKLIPRAIDWFTG
AN8863.1  	VKITVPTESFFNFFAPPQPPADDDD-TVATDIEERLELDYQLGEDIKEKLIPRAIDWFTG
          	** ***********:**:.*::**:  . :***:**************************

MG06924.1 	EALAFEEMDEDDLE-DDFEDEDDDD--DLSEDHD----DDEESDDD~D0D~D-ASKPKQE
NCU01438.1	EALAFEELDEEDFEDAEFDDEDDEDDEDASEDQD----DEDESDED~D0E~D-GSKPKQE
FG07467.1 	EALAFEEISDDELDGADFDDDDDEDEDDLSEEND----DEEESDDD0D~E~--SGKPKQE
AN8863.1  	EALQFEELG-DDMDPDEFDEFDDEDDEDDDEDEDGRGSDQDVDDSD~E~E0DGTSKPKKE
          	*** ***:. ::::  :*:: **:*..* .*:.*. .:*:: .*.* : : .. .***:*

MG06924.1 	AAECKQS
NCU01438.1	AAECKQS
FG07467.1 	AAECKQS
AN8863.1  	AAECKQS
          	*******
```
